# Propofol effects in rodent models of traumatic brain injury: a systematic review

**DOI:** 10.2478/abm-2021-0032

**Published:** 2021-12-30

**Authors:** Riyadh Firdaus, Sandy Theresia, Ryan Austin, Rani Tiara

**Affiliations:** Department of Anesthesiology and Intensive Therapy, Faculty of Medicine, Universitas Indonesia, Dr. Cipto Mangunkusumo Hospital, Jakarta 10430, Indonesia

**Keywords:** anesthetics, brain injuries, traumatic, neuroprotection, propofol, Rodentia

## Abstract

**Background:**

Traumatic brain injury (TBI) causes high mortality and disability worldwide. Animal models have been developed to explore the complex processes in TBI. Propofol is used to manage head injuries during surgical intervention and mechanical ventilation in patients with TBI. Many studies have investigated the neuroprotective effect of propofol on TBI. However, other studies have shown neurotoxic effects.

**Objectives:**

To review systematically the literature regarding the neuroprotective and neurotoxic effects of propofol in rodent models of TBI.

**Methods:**

Data from rodents as models of TBI with propofol as one of the intervention agents, and/or comparing the neuroprotective effects of propofol with the other substances in rodent models of TBI, were obtained from PubMed, EBSCO Host, and ProQuest databases. The PRISMA 2020 statement recommendations were followed and research questions were developed based on PICOS guidelines. Data was extracted from the literature using a standardized Cochrane method.

**Results:**

We analyzed data from 12 articles on physiological changes of experimental animals before and after trauma, the effects of propofol administration, and the observed neurotoxic effects. The effects of propofol administration were observed in terms of changes in traumatic lesion volume, the release of antioxidants and inflammatory factors, and the neurological function of rodent models of TBI.

**Conclusion:**

Propofol has neuroprotective and neurotoxic effects via several mechanisms, and various doses have been used in research to determine its effects. The timing of administration, the dose administered, and the duration of administration contribute to determine the effect of propofol in rodent models of TBI. However, the doses that produce neuroprotective and neurotoxic effects are not yet clear and further research is needed to determine them.

Traumatic brain injury (TBI) causes high mortality and disability worldwide [1]. Data from 1990 to 2016 show 27 million TBI cases, with an average incidence of 369 cases per 100,000 population every year worldwide. This number might be lower than the actual number of cases as the incidence and distribution of TBI across nations remain unknown [2, 3]. TBI is an injury to the brain that is usually caused by a bump, blow, or jolt to the head from blunt trauma or penetrating injury, such as from a motor vehicle accident or a gunshot [4]. TBI consists of primary and secondary injuries. Primary brain injury (focal and diffuse) results from mechanical injury at the time of the trauma, whereas secondary brain injury is caused by the physiological responses to the initial injury, leading to release of neurotoxic substances and neuronal cell death [5, 6]. Hypotension and systemic hypoxia can exacerbate the secondary injury and are strongly associated with higher mortality risks [7]. To explore further the complex processes that occur in TBI in humans, several models of TBI have been developed using laboratory animals [6].

Propofol (2,6-bis(propan-2-yl)phenol) is an anesthetic agent, which is often used intravenously in the management of TBI, including for surgical intervention or sedation in an intensive care unit [8]. Propofol has advantages of minimal side effects and a short onset of drug activation and duration, along with relatively facile control of the depth of anesthesia. Apart from its anesthetic effects, propofol has properties as an anxiolytic, immunomodulator, analgesic, antiemetic, and inhibits platelet aggregation among other effects [9]. Propofol has shown neuroprotective effects due to its anti-oxidant activity and its ability to decrease brain metabolic rate, redistribute cerebral blood flow, suppress glutamates during ischemic events, and regulate apoptosis-related proteins [10].

Although there are many studies investigating the neuroprotective effect of propofol in TBI, other studies have produced discordant results. Propofol has shown neurotoxic effects on the brain, which caused increased mortality, worsened neurobehavioral outcomes, and reduced hippocampal neurogenesis [11]. To our knowledge, no systematic review has addressed this issue to date. Here, we sought to identify systematically studies from the literature to determine the effect of propofol in terms of physiological parameters, post administration effects, and dose used to determine the dominant effect of propofol in rodent models of TBI.

## Methods

This systematic review was conducted according to the recommendations of the preferred reporting items for systematic reviews and meta-analysis with a PRISMA 2020 statement checklist [12]. The research question was “In studies in rodent models of TBI, does propofol exhibit neuroprotective effects?”. This research question was constructed following the PICOS guidelines [13].

### Study selection

We selected articles that met the inclusion criteria, which were a trial using a rodent model of TBI, with propofol as one of the intervention agents. We also included articles comparing the neuroprotective effects of propofol with other agents in rodent models of TBI. We excluded articles that were not available in full-text form and studies that were not published in English.

### Data sources and searches

Relevant publications were identified using PubMed, EBSCO Host, and ProQuest search engines without restrictions or filters of publication date or language. The search keywords included MeSH: propofol, TBI, traumatic brain injury, neuroprotection, neuroprotective agents, and rodents. In addition to the MeSH keywords, we also added 2 other keywords not listed in the MeSH: neurotoxicity and craniocerebral trauma. A reference search from selected articles was conducted manually. All study searches were completed by March 1, 2021.

### Data extraction and result synthesis

Data was extracted from the selected studies by 2 reviewers independently using Cochrane standardized data extraction form [14]. Data extracted from each study included year of publication, study design, study objectives, number of experimental groups, analytical methods, stated limitations and recommendations, and the study conclusions. A validity assessment was also conducted using the Cochrane risk-of-bias tool [15]. All data extracted from the selected studies were compiled into a table and explained qualitatively.

## Results

### Study identification

**Figure 1:**
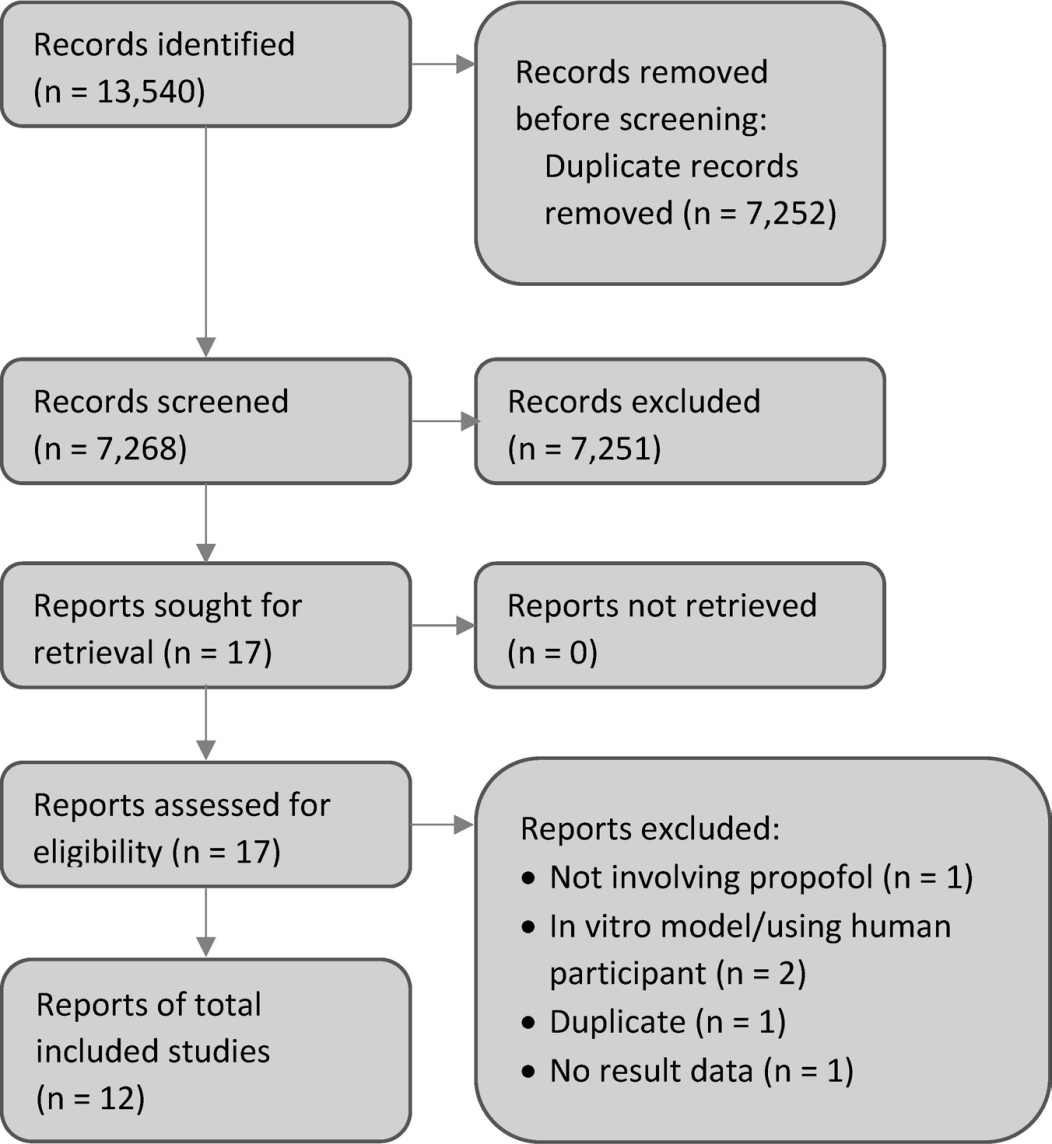
Flow diagram of the study selection process.

Based on the search strategy described above, 13,540 studies were found. After duplicates were removed, 9,286 studies remained, and prespecified criteria excluded 2,018 more studies, leaving 7,268. Then, these studies were screened by reading the titles and abstracts of each study, and 17 relevant studies were obtained. Of the 17 studies, we evaluated eligibility and feasibility of data extraction with a standardized form and found 12 that met the requirements for data extraction and further analysis (**Figure 1**).

### Selected study quality

The assessment was conducted using the inclusion criteria. Overall, the study findings show a large variety of trauma types and methods used to evaluate the effects of propofol. The research methods and designs used were heterogeneous. Most of the researchers did not explain the bias that might occur in their respective studies [16, 17, 18, 19]. Only 3 articles described the bias that could affect the results of the study they reported [20, 21, 22]. Moreover, only one study used investigators blinded to the treatment during the intervention process and the assessment of intervention results [23].

## Main results

### Physiological changes

Characteristics of the included studies are shown in **Table 1**. Of the 12 studies included in the present research, 8 assessed the physiological parameters of experimental animals. Mean arterial pressure (MAP), heart rate, pH, PaCO_2_, and PaO_2_ were not found to be significantly different in 4 studies, both in the pre- and post-trauma evaluation, and the evaluation in each experimental group. Another 3 articles did not state whether there was a change in physiological factors during the experiment, and the remaining article reported differences found in physiological parameters that were affected by the dose of propofol administered [16, 18, 19, 20, 22, 23, 24, 25, 26]. Apart from the previously mentioned physiological parameters, studies by Sebastiani et al. [23], Statler et al. [24], and Thal et al. [25] also assessed core temperature, brain tissue temperature, hematocrit, and serum glucose levels in rats or mice during the experimental procedure, all of which were within normal limits, and there were no differences found between groups.

**Table 1:** Summary of study characteristics

**No.**	**Reference**	**Objectives**	**Animals/groupings**	**Type of injury**	**Analysis**	**Limitations/recommendations**	**Conclusion**
1	Menku et al. [20]	Effect of propofol, propofol-citicoline combination on LP after head injury	39 adult, male, Swiss Albino rats, 250–300 g Divided into 3 groups (n = 13 each): Control group Propofol group Propofol + citicoline group	Moderate diffuse brain injury	Physiological parameters Heart rate MAP Hematocrit pH PaCO_2_ PaO_2_ MDA level SOD level GPx level	Combined use of therapeutic agents is more useful due to the synergetic effects	Effects of propofol on the activity of antioxidant enzyme system were stronger than its effect on LP
2	Ding et al. [17]	Therapeutic effects of propofol on brain edema following TBI and the modulating effect of propofol on AQP-4 expression	Male, Sprague Dawley rats, 250–300 g Divided into 4 groups: TBI+P (n = 10) P group (n = 10) TBI group (n = 10) Sham-operation group (n = 8)	Weight-drop injury	Brain edema AQP-4 gene expression AQP-4 level IL-1β and TNF-α expression Cultured astrocytes (NFκB and p38)	Control of brain edema and reduced neuroinflammation following TBI. May prove clinically useful in managing acute TBI as a multifunctional neuroprotective agent.	Propofol treatment soon after TBI attenuates the development of edema and inhibits AQP-4 overexpression in rat models of TBI. Propofol modulates acute AQP-4 expression by attenuating IL-1β and TNF-α expression and inhibiting IL-1β and TNF-α-induced AQP4 expression
3	Ma et al. [21]	Role of NLRP3 and the effect of propofol to inhibit the NLRP3 inflammasome activation, which probably mediates the protective activity of propofol against the secondary injury following bTBI	Male SPF Sprague Dawley rats (220–300 g) Divided into 6 groups (n = 8 each): Normal group bTBI-12 h and 24 h group rats were harvested at 12 h and 24 h after injury (n = 8, each subgroup) bTBI-12 h and 24 h group treated with propofol (n = 8, each subgroup) bTBI DMSO control group	Blast-induced TBI (bTBI)	NLRP inflammasome mRNA expression TXNIP Caspase-1-p20 Cytokine level SOD MDA NSE	-	Overactivation of the NLRP3 inflammasome in the cerebral cortex may be involved in neuroinflammation during the secondary injury of bTBI in rats. Propofol might relieve the inflammatory response and attenuate brain injury by inhibiting ROS and reluctant depressing NLRP3 inflammasome activation and proinflammatory cytokine expression
4	Eberspächer et al. [16]	Effect of EEG-targeting low- and high-dose propofol infusion on acute histopathological damage in rats subjected to a moderate CCI	39 male Sprague Dawley rats (400 ± 50 g) In 4 groups: CCI/lowprop (n = 10) CCI/highprop (n = 10) CCI/halo (n = 10) Sham/halo (n = 9)	CCI	Physiological evaluation MAP Heart rate Hb PaO_2_ PaCO_2_ pH Respiratory rate Glucose Core and pericranial temperature Lesion volume Eosinophilic cells Activated caspase-3	Rupturing vessels during the impact that may lead to cortical and subcortical hemorrhage exacerbating the primary injury	EEG-targeted low- and high-dose propofol infusion for 6 h after CCI did not affect lesion volume or the number of eosinophilic cells in the hippocampus
5	Kahveci et al. [18]	Compared cerebral protective effects of isoflurane and propofol, when used in combination with moderatehypothermia (33–34 °C) in rats with TBI	16 female Wistar rats (275–350 g) 2 groups (n = 8 each): Isoflurane group Propofol group	Diffuse impact-acceleration	Physiological data MAP pH PaCO_2_ PaO_2_ Hematocrit	Did not control the MAP during the study period. Investigation is needed to define the neuroprotective dose range of propofol in both experimental and human head injury	Propofol may be a better choice than isoflurane for use in combination with moderate hypothermia as treatment for head injury
6	Liu et al. [19]	Investigated the neuroprotective effects of propofol in rats following TBI, determined by neurological severity scores and expression of proinflammatory cytokines in the injured cortex	212 adult male Sprague Dawley rats (200 ± 20 g) Divided into 4 groups (each group, n = 53): Sham control group TBI group TBI+intralipid group TBI+propofol group	Weight-drop injury	Neurological functional outcome (NSS test) IL-1β, IL-6 and TNF-α mRNA expression IL-1β, IL-6 and TNF-α protein level	-	Cytokines, IL-1β, IL-6 and TNF-α play key roles in the pathophysiology of neuroinflammation in the delayed phase of TBI, and propofol could reduce the neurological impairment induced by TBI via suppression of inflammatory reaction
7	Luo et al. [22]	Anti-inflammatory effects of propofol after lateral fluid percussion TBI	Adult male Sprague Dawley rats (300–340 g) Divided into 4 groups: Isoflurane-TBI Isoflurane-sham Propofol-TBI Propofol-sham No data on the number of rats per group	Lateral fluid percussion trauma	Animal physiology Cognitive recovery (MWM test) Lesion size after TBI Neuronal loss in cortex Microglial activation Propofol reduces lipopo-lysaccharide-stimulated primary microglia activation and neurotoxicity NADPH oxidase activity Expression of gp91phox and p22phox	General anesthetics before and during the delivery of TBI, which do not ideally reflect clinical situations	Effects of propofol on microglial activation substantial
8	Öztürk et al. [27]	Effects of propofol and erythropoietin (Epo) on brain injury caused by oxidative stress and antioxidant properties of these agents after CHI	60-d-old female Wistar albino rats (200–250 g) Divided into 5 groups: Control (n = 4) CHI (n = 6) Epo (n = 6) Propofol (n = 6) Epo + propofol (n = 6)	CHI	Biochemical analysis SOD activity CAT activity MDA level XO level NO level	Lack of functional outcome measures	Epo and propofol decreased oxidative stress by decreasing MDA and NO level in brain tissue after CHI. However, combination of Epo and propofol has no significant beneficial advantage over Epo or propofol alone
9	Sebastiani et al. [23]	Delayed single-bolus propofol applications at the peak of p75NTR p75expression after experimental TBI	213 adult C57BL/6N and 15 NGFR–deficient mice 6 groups (propofol 6 h, propofol 24 h, vehicle 6 h, vehicle 24 h, NaCl 0.9% 6 h, NaCl 0.9% 24 h)	CCI	Physiological data (each group, n = 11) Delayed post-traumatic propofol sedation enhances brain lesion volume (each group, n = 10) Post-traumatic propofol sedation induces αII-spectrin degradation and reversibly lowers c-Fos levels, but does not modulate caspase-3 activation (each group, n = 5) Post-traumatic propofol sedation impairs long-term motor function (each group, n = 13) Expression of p75NTR is increased after experimental TBI (each group, n = 9–10) Propofol-mediated increase in brain damage after CCI is attenuated by pharmacological inhibition of p75NTR and in p75NTR-deficient animals (each group, n = 11 adult C57BL/6N; n = 5 NGFR–deficient mice) Propofol-mediated neuronal cell death is attenuated in NGFR^−^/^−^ mice (each group, n = 5) Propofol sedation inhibits proteolytic cleavage of proapoptotic p75NTR ligand proBDNF after TBI (each group, n = 5)	Not stated	Propofol sedation after acute brain lesion can have a deleterious impact and implicates a role for the pro-brain-derived neurotrophic factor-p75NTR p75 pathway. Propofol and other compounds with GABA receptor activity are frequently used in patients with acute brain pathologies to facilitate sedation or surgical and interventional procedures
10	Statler et al. [24]	Degree of neuroprotection provided by 7 anesthetics or sedatives (diazepam, fentanyl, isoflurane, ketamine, morphine, pentobarbital, and propofol) in a standard model of TBI	81 adults, male Sprague Dawley rats (300–440 g) Divided into 9 groups of n = 9 (diazepam, fentanyl, isoflurane, ketamine, morphine, pentobarbital, propofol, none, and sham)	CCI	MAP during experiment Time to extubation PaCO_2_ and PaO_2_ post TBI Motor function Cognitive testing Histological analysis	Compared various anesthetics/sedatives, does not allow precise assessment of the effects of individual anesthetics/sedatives when administered in the absence of isoflurane pretreatment (isoflurane may be neuroprotective). Difficulty comparing the potency of inhalational and intravenous anesthetics (equipotent doses, based on the traditional concept of MAC, may not have been achieved)	Medications commonly used in clinical TBI, narcotics and propofol, produced the poorest outcomes. By contrast, isoflurane, which is frequently used in experimental TBI, was associated with the best outcomes
11	Thal et al. [25]	To establish the influence of propofol on endogenous neurogenesis and functional recovery after TBI, rats were sedated with propofol (36 or 72 mg/kg/h) either during (0.5 h before until 1.5 h after CCI) or 2 h after TBI (3 h propofol sedation with 32 mg/kg/h (low dose (LD)) or 72 mg/kg/h (high dose (HD))	116 male Sprague Dawley rats (280–330 g) divided into 5 groups in study A and 5 groups in study B (various number of mice per group) Study A: Naïve (n = 10) Sham36 (n = 11) Sham72 (n = 12) CCI36 (n = 10) CCI72 (n = 11) Study B: Naïve (n = 10) Primary lesion (n = 11) CCI + 0 (n = 11) CCI + LD (n = 13) CCI + HD (n = 17)	CCI	Mortality, physiological variables, lesion volume Study A: Influence of peritraumatic propofol infusion on neurogenesis, dentate gyrus volume, and neurofunctional outcome Study B: Influence of post-traumatic propofol on neurogenesis and neurofunctional outcome	Use of BrdU to label newly generated cells in rodents, proliferation induced by ischemia reaches a maximum after 7–11 d. Study only following the subject for 7 d, proliferation after day 7 following CCI will not be detected and is missed	Propofol infusion significantly attenuated post-traumatic neurogenesis, deteriorated neurofunctional recovery, and increased 28-d mortality. Potential negative effect of propofol after acute brain injury, especially when it is applied 2 h after CCI
12	Yu et al. [26]	Propofol could attenuate LP, calpain-induced CRMP2 degradation, and brain injury after TBI	70 adult male Sprague Dawley rats (220–270 g) 7 groups of 9 rats (sham control, TBI, TBI + propofol 1 h, TBI + propofol 2 h, TBI + propofol 4 h, TBI + UE83836E, TBI + fat emulsion)	Unilateral moderate CCI injury	Hemodynamics and arterial blood gas analysis Propofol attenuates post-traumatic oxidative damage Propofol reduces calpain-mediated αII-spectrin breakdown Propofol reduces the proteolysis of collapsin response mediator protein-2 after TBI Propofol protects neurons from TBI-induced apoptosis	Propofol ameliorates oxidative stress, suppresses calpain activation and CRMP2 proteolysis and reduces apoptosis, propofol likely may interact with other signaling pathways that may or may not be involved in post-TBI peroxidation, calpain activation and CRMP2 degradation. The samples at 24 h following TBI: can only make conclusions regarding the transient neuroprotective effects of propofol postconditioning in TBI rats. Long-term and dose-dependent effects of propofol must be investigated further. Additional time points are also needed to determine the best therapeutic window for propofoladministration. Extrapolating the therapeutic regimen of propofol for TBI rats to patients with moderate TBI should be performed cautiously and requires further clinical research.	Propofol postconditioning alleviates calpain-mediated CRMP2 proteolysis and provides neuroprotective effects following moderate TBI potentially by counteracting LP and reducing calpain activation

AQP-4, aquaporin-4; BrdU, bromodeoxyuridine; bTBI, blast-induced traumatic brain injury; CAT, catalase; CHI, closed head injury; CRMP2, collapsin response mediator protein-2; CCI, controlled cortical impact; DMSO, dimethyl sulfoxide; EEG, electroencephalogram; GABA, γ-aminobutyric acid; GPx, glutathione peroxidase; HD, high-dose propofol; IL, interleukin; IL-1β, interleukin-1β; LP, lipid peroxidation; LD, low dose; MDA, malondialdehyde; MAP, mean atrial pressure; MAC, minimum alveolar concentration; MWM, Morris Water Maze; NGFR, nerve growth factor receptor; NSE, neuro-specific enolase; NO, nitric oxide; NLRP3, NOD-like receptor family pyrin domain containing 3; NFκB, nuclear factor kappa B; NSS, neurological severity score; p75NTR p75, neurotrophin receptor; PaCO_2_, partial pressure of carbon dioxide; PaO_2_, partial pressure oxygen; proBDNF, prodomain brain-derived neurotrophic factor; P, propofol; ROS, reactive oxygen species; SOD, superoxide dismutase; TXNIP, thioredoxin interacting protein; TBI, traumatic brain injury; TNF-α, tumor necrosis factor α; XO, xanthine oxidase.

### Effects of propofol administration on lesion volume

A significant reduction in lesion volume after administration of propofol was found in 2 studies. The volume reduction effect is stronger when propofol is given early [17, 22]. No change in lesion size after administration of propofol was found in another 2 studies, even when low dose (20 mg) and high dose (30 mg) of propofol were administered to investigate a possible dose-dependent effect [16, 25]. Sebastiani et al. [23] found that early propofol administration (6 h post injury) did not substantially influence lesion volume. Meanwhile, delayed propofol administration (24 h after exposure to trauma) was associated with significant increase of lesion volume in a mouse group with propofol sedation compared with a control group. Statler et al. [24] found propofol increases lesion size in adult rats, as do other anesthetic agents. There was no lesion size data for the remaining 6 studies.

### Effects of propofol administration on antioxidant release

From the studies analyzed, we found 3 that assessed the levels of superoxide dismutase (SOD) and malondialdehyde (MDA) in rats administered propofol and found an increase in SOD levels and a decrease in MDA levels in brain tissue [20, 21, 27]. However, Öztürk et al. [27] did not find a significant difference in SOD levels. In addition to SOD and MDA levels, Öztürk et al. [27] also measured the levels of xanthine oxidase (XO), levels of nitric oxide (NO), and catalase activity (CAT) in brain tissues. CAT levels were not found to be significantly different between the groups, while XO and NO levels were lower in the propofol group. The difference in NO levels was significant, while XO levels were not. Ma et al. [21] measured glutathione (GSH) levels, another indicator in assessing the oxidative stress response, which were elevated after propofol administration.

### Effect of propofol administration on the release of inflammatory factors

Inflammatory factors such as interleukin-1β (IL-1β) and tumor necrosis factor-α (TNF-α) are released spontaneously in brain tissue after TBI, but propofol can reduce their levels. Ding et al. [17] specifically described the release of IL-1β and TNF-α and the effect of propofol on pyrrolidine dithiocarbamate (PDTC) activation pathway as an inhibitor of nuclear factor kappa B (NFκB) and SB203580 as an inhibitor of P38 mitogen-activated protein kinases (p38/MAPK), which was used to assess specific pathways of aquaporin-4 (AQP-4) expression. Propofol acts as an inhibitor of both the NFκB and p38/MAPK pathways. qRT-PCR analysis found that propofol clearly inhibits the expression of the AQP-4 mRNA in vivo, which is mediated by NFκB. This inhibition by propofol is influenced by the dose given and the time of administration. The explanation for the timing of administration effectiveness relates to activation of acute transcription of AQP-4 after the onset of TBI [17]. A decrease in the levels of the NOD-like receptor family pyrin domain containing 3 (NLRP3) inflammasome occurred after propofol administration in rats. The levels of IL-1β and TNF-α in the cerebral cortex tissue increased in the first 12 h and 24 h after trauma, while propofol administration at the time of trauma decreased release of these inflammatory factors [21]. After the administration of propofol, the expression of IL-1β, IL-6, and TNF-α mRNA decreased inflammation in rats compared with an experimental group without propofol administration. After the administration of propofol, the protein levels of the 3 cytokines were significantly decreased [19]. Based on these 3 studies, we infer that propofol exerts neuroprotective effects by reducing inflammatory factors [17, 19, 21].

### Neurotoxic effects of propofol

Sebastiani et al. [23] found propofol had neurotoxic effects through calpain activity increase as seen from the quantification of αII-spectrin degradation results. Calpain or calcium-regulated cysteine protease promotes cell death. The amount of αII-spectrin degradation was not affected by propofol in the first hour after trauma, but increased significantly in the following 6 h. Moreover, propofol administration expanded lesions because of TBI via a neurotrophic receptor pathway by mediating an increase of pro-brain-derived neurotrophic factor-p75 levels. Yu et al. [26] found propofol had the opposite effect. After propofol administration up to 4 h after TBI, the quantity of αII-spectrin degradation products was significantly lower in the pericontusional cortex of rats at 24 h after TBI compared with vehicle control, although we note the use of chloral hydrate in their model which is unacceptable for anesthesia.

### Effects of propofol on neurological function

Neurological function has been assessed based on the motor and cognitive functions of rat models. Motor function in rats was assessed by beam balance and beam walking tests. The rats from both propofol-administered and control groups showed disturbances in motor function after injury. However, Statler et al. [24] showed that rats in a group given propofol showed more severe impairment than the control group. Thal et al. [25] also showed that motor impairment was more severe in rats administered larger doses (72 mg/kg/h) of propofol. Cognitive function was assessed using the Morris Water Maze (MWM) and Barnes maze tests [24, 25]. In the MWM, rats in the propofol group showed slower learning ability than a control group [24]. The Barnes maze test results varied greatly between rats; and the investigators were unable to show any significant differences between groups [25]. Lou et al. [22] found no significant changes on MWM assessment on days 14 and 17 after TBI followed immediately by propofol administration.

### Propofol doses

The doses of propofol administered in the various studies differed widely, as did the route of drug administration. In 5 studies, the dose range for bolus intraperitoneal administration of propofol ranged from 50 mg/kg to 100 mg/kg [17, 19, 20, 21, 27]. However, standard administration of propofol is by intravenous infusion. In another 5 studies, intravenous administration with doses varying from 10 mg/kg/h to 85 mg/kg/h were used. Yu et al. [26] used intravenous bolus. It is not clear which dose of propofol caused neuroprotective effects nor is it clear which dose causes neurotoxic effects [18, 23, 24, 25, 26].

## Discussion

The effects of propofol on brain tissue after TBI can be neuroprotective or neurotoxic and are not yet fully understood. Ethical considerations are a factor that makes the effects of propofol difficult to study directly in humans. In trials, it is not possible to observe directly the processes that occur in the patient's brain histologically, biochemically, or immunohistochemically. Although its effects can be neuroprotective or neurotoxic, propofol is an anesthetic agent commonly used in intensive care units because of its high safety profile. Moreover, propofol is superior to benzodiazepines or other drugs because it carries less risk of causing respiratory system suppression and has a short sedation duration, making it possible to carry out a periodic neurological assessment [28]. Therefore, we conducted a systematic review to investigate the physiological changes, the changes of the traumatic lesion size, and the neuroprotective or neurotoxic mechanisms arising from propofol administration to rodents. Rodents are commonly chosen for experimental models of TBI as they are affordable, small, and have standardized physiological parameters [29].

The neuroprotective effects of propofol in the rodent models of TBI are associated with antioxidants, potentiation of γ-aminobutyric acid type A (GABA_A_) receptors, which mediate inhibition of synaptic transmission and inhibition of glutamate release. Propofol also modulates various aspects of the host inflammatory response. It reduces the release of proinflammatory cytokines, alters NO expression, interferes with the function of monocytes and neutrophils, and has the potential to counteract free radicals [8]. Moreover, propofol administration combined with moderate hypothermia can significantly reduce intracranial pressure and increase cerebral perfusion pressure. Administration of propofol before weight-drop injury demonstrated a neuroprotective effect in rats by modulating NO synthesis and reducing lipid peroxidation (LP) [30].

Propofol is commonly used in patients with acute brain injury to facilitate sedation during surgery. In the present study, we reviewed 12 studies in rodents to identify the neuroprotective or neurotoxic effects of propofol. We found 8 studies indicating propofol having neuroprotective effects through various mechanisms. We also found studies indicating neurotoxic effects from propofol administration, which were induction of neuron cell death through the neurotrophic receptor pathway by mediating pro-brain-derived neurotrophic factor-p75, deteriorating motor function, interfering with the neurogenesis process, and increased mortality.

### Physiological changes

Propofol administration often causes side effects in the form of hypotension. Hypotension is categorized as a mild side effect when compared with the advantages of propofol which can maintain cerebral perfusion pressure in patients with TBI [28]. Propofol did not show any significant effect on the physiological parameters of rodents that underwent experimental TBI, either before or after the trauma [16, 18, 20, 22, 23, 24, 25, 26]. These findings are consistent with data on propofol in humans, which shows a good safety profile [28, 31, 32]. Statler et al. [24] explored the duration from trauma to extubation, and found rats administered propofol required a significantly longer time to extubation than those administered isoflurane, rats in a sham group, and rats that did not receive additional anesthetic agents.

### Propofol effects

Controlled cortical impact (CCI) type trauma in rodent models of TBI produced an increase in the volume of brain lesions after 24 h [33]. Histological assessment by Luo et al. [22] found a decrease in the volume of lesions after administration of propofol compared with that after administration of isoflurane. However, Eva et al. [16] found that the use of propofol did not affect lesion volume when compared with halothane as the anesthetic agent. The difference in the results might be a consequence of differences in the weight of the rats and the type of head injury. The overall primary injury lesion volume found by Thal et al. [25] was 23.8 ± 7.4 mm^3^, although not all data were shown. This study found no significant differences in lesion volume associated with propofol administration. Propofol administration within 24 h post trauma has shown an effect of increasing the lesion volume. However, the administration of propofol within 6 h produced no significant increase of lesion volume [23].

Secondary injury in TBI results in many pathophysiological effects, such as ion imbalance, inflammation, apoptosis, endoplasmic reticulum stress, and oxidative stress [34]. Oxidative stress itself produces reactive oxygen species (ROS) and causes a redox imbalance, producing LP, nucleic acid oxidation, and DNA damage leading to neuronal damage and neuron cell death [35]. Lipid peroxidase activity is assessed by the level of MDA, and the changes in cerebral cortex endogenous antioxidants post TBI can be assessed based on levels of SOD and GSH. As expected, propofol administration increased the activity of SOD and GSH, which would decrease MDA levels in the process of oxidative stress [21]. In the present review, we found propofol administration reduced oxidative stress by attenuating microglia activation, MDA, and NO levels.

The inflammatory response after TBI will activate microglia and astrocytes, release inflammatory mediators in the brain, and mobilize peripheral immune cells (e.g., leukocytes and monocytes). Biomarkers commonly used for anti-inflammatory and proinflammatory molecules are TNF-α, IL-1β, IL-6, IL-8, and IL-10 [36]. Propofol can decrease neurological damage by suppressing the inflammatory reaction, with the inflammatory cytokines most affected by propofol administration being IL-1β and TNF-α [17, 19, 21]. Overactivation of the NLRP3 inflammasome after trauma in the cerebral cortex affects the neuroinflammatory processes leading to secondary trauma. Propofol can mitigate the inflammatory response, reduce brain damage by inhibiting ROS production and depress activation of the NLRP3 inflammasome, and influence the expression of proinflammatory cytokines [21]. The glymphatic system plays a role in directing cerebrospinal fluid to enter the brain through the periarterial space, into the interstitium via AQP-4. After TBI, the function of the glymphatic pathway is impaired and decreases by up to 60% [36]. Administration of propofol immediately after TBI reduces edema and will inhibit the overexpression of AQP-4 [17].

Apart from the direct effect of propofol on neurotrans-mission, propofol also results in impaired mitochondrial function in neurons, which is responsible for neurotoxicity and postoperative brain dysfunction. During the anesthesia, propofol inhibits electrophysiological processes that can inhibit ATP requirements [37]. Sebastiani et al. [23] demonstrated that immediate administration of propofol after acute injury produces an adverse effect mediated by pro-brain-derived neurotrophic factor-p75. By contrast, propofol also increases calpain activity as seen from the high degradation result of αII-spectrin by the calpain-dependent proteolysis mechanism. Increased degradation of αII-spectrin by calpain-dependent proteolysis is thought to be a mechanism for propofol-induced neurotoxicity in the developing brain that results in structural changes, changes in synaptic plasticity, and activation of the cell death process [23]. By contrast, Yu et al. [26] did not find a neurotoxic effect of propofol. The significantly lower level of αII-spectrin degradation in the propofol group suggested that propofol was active and neuroprotective in the calpain activation pathway in TBI.

A strong adverse effect of propofol administration is a decrease in the post-traumatic neurogenesis process, especially if it is administered within a few hours post trauma. The brain will undergo physiological changes to maintain and enhance neurogenesis during TBI, whereas the process will be hindered by propofol infusion. Neurogenesis has an important role in memory function. The inhibition of neurogenesis results in a decrease in cognitive and motor function in both humans and rats [24, 25]. Statler et al. [24] showed that rats given propofol experienced more severe impairment than a control group [24]. Thal et al. [25] also showed that motor impairment was more severe in rats receiving propofol in larger doses. Impaired cognitive function on MWM assessment as an indicator of hippocampal damage can be measured by counting the number of neurons in the region. Luo et al. [22] found no significant difference of propofol administration in improving cognitive abilities according to a MWM test, as consistent with the state of TBI, which induces neuron cell death.

We did not limit the present review by the type of TBI. Therefore, the conclusions drawn are the result of general analysis and are not limited to particular types of TBI. Moreover, some of the literature analyzed reported propofol administration before and during the trauma (not only after the trauma process occurred). This may have biased conclusions on assessing the effects of propofol, which is most commonly used after trauma occurs.

## Conclusion

Propofol has protective effects in rodent models of TBI via several mechanisms, including through GABA_A_ receptor-mediated inhibition of synaptic transmission, decreased inflammatory reaction, which delays neurological damage, decreases oxidative stress, and inhibits glutamate release. Various doses have been used in research models of TBI to determine the effect of propofol; however, the doses required to obtain neuroprotective and neurotoxic effects are not yet clear. The timing of propofol administration after TBI, the dose of propofol administered, and the duration of propofol administration contribute to determining the effect of propofol on TBI. Further research is needed to determine the dose range effecting neuroprotection and neurotoxicity to guide clinical research in humans.
